# Insights into the Role of Substrates on the Interaction between Cytochrome *b*_5_ and Cytochrome P450 2B4 by NMR

**DOI:** 10.1038/srep08392

**Published:** 2015-02-17

**Authors:** Meng Zhang, Stéphanie V. Le Clair, Rui Huang, Shivani Ahuja, Sang-Choul Im, Lucy Waskell, Ayyalusamy Ramamoorthy

**Affiliations:** 1 Department of Chemistry and Biophysics, University of Michigan, Ann Arbor, Michigan 48109-1055, USA; 2 Department of Anesthesiology, University of Michigan, and VA Medical Center, Ann Arbor, Michigan 48105, USA

## Abstract

Mammalian cytochrome *b*_5_ (cyt *b*_5_) is a membrane-bound protein capable of donating an electron to cytochrome P450 (P450) in the P450 catalytic cycle. The interaction between cyt *b*_5_ and P450 has been reported to be affected by the substrates of P450; however, the mechanism of substrate modulation on the cyt *b*_5_-P450 complex formation is still unknown. In this study, the complexes between full-length rabbit cyt *b*_5_ and full-length substrate-free/substrate-bound cytochrome P450 2B4 (CYP2B4) are investigated using NMR techniques. Our findings reveal that the population of complexes is ionic strength dependent, implying the importance of electrostatic interactions in the complex formation process. The observation that the cyt *b*_5_-substrate-bound CYP2B4 complex shows a weaker dependence on ionic strength than the cyt *b*_5_-substrate-free CYP2B4 complex suggests the presence of a larger fraction of steoreospecific complexes when CYP2B4 is substrate-bound. These results suggest that a CYP2B4 substrate likely promotes specific interactions between cyt *b*_5_ and CYP2B4. Residues D65, V66, T70, D71 and A72 are found to be involved in specific interactions between the two proteins due to their weak response to ionic strength change. These findings provide insights into the mechanism underlying substrate modulation on the cyt *b*_5_-P450 complexation process.

Cytochrome P450s (P450s) are a ubiquitous superfamily of monooxygenases responsible for the metabolism of numerous endogenous and exogenous compounds, including over 50% of marketed pharmaceuticals^1^,^2^,^3^. Mammalian P450 and its redox partners, cytochrome P450 reductase (CPR) and cytochrome *b*_5_ (cyt *b*_5_), are membrane-bound proteins, each comprised of a large soluble domain and a single α-helical transmembrane domain^4^. To carry out the well-known oxidation of non-activated hydrocarbon molecules, P450 requires two electrons to be sequentially delivered from its redox partners^5^. CPR is capable of donating both electrons, while cyt *b*_5_ is only able to provide the second electron to oxyferrous P450 but not the first electron to ferric P450 due to its high redox potential^6^,^7^,^8^,^9^. It has been proposed that electron transfer proteins follow a two-step model for complex formation: first, the formation of an ensemble of encounter complexes in which the two partners adopt different orientations relative to one another; second, the formation of well-defined complex(es), referred to as the stereospecific complex(es), in which electron transfer can occur^10^,^11^. Long-range, non-specific electrostatic interactions have been found to be the main driving forces behind encounter complex formation, while it is the specific, short-range interactions (*e.g.* hydrogen bonds, salt bridges and hydrophobic interactions) that hold the stereospecific complex(es) together^11^,^12^,^13^,^14^,^15^. It has been reported that encounter complexes exhibit stronger dependence on ionic strength than the stereospecific complex^16^.

Cyt *b*_5_ has been shown to stimulate, inhibit, or have no effect on the activity of P450s, both *in vitro* and *in vivo*, in humans and genetically engineered mice^9^,^17^,^18^,^19^. Although our understanding of the novel effects of cyt *b*_5_ on P450 catalysis is incomplete, significant progress has been made towards understanding its mechanism of action. Its stimulatory effects are due to its ability to generate the active oxidizing species of P450 more quickly than CPR^20^. The more rapid rate of catalysis in the presence of cyt *b*_5_ also allows less time for side product formation, thereby increasing the utilization of NADPH for product formation. Cyt *b*_5_ inhibits P450 activity by competing with CPR for a binding site on the basic proximal surface of P450^8^,^9^. No effect of cyt *b*_5_ is observed when these two opposite effects cancel one another. It has also been reported that cyt *b*_5_ exhibits different stimulatory effects on substrate turn-over depending on the substrate under investigation^17^,^21^. It has also been found that a higher affinity between cyt *b*_5_ and P450 could be achieved in the presence of substrates^22^. A solution NMR study on truncated P450 also revealed substrate modulation of the interactions between cyt *b*_5_ and P450^23^. Our previous work on the full-length cytochrome P450 2B4 (CYP2B4) showed that the substrate 3,5-di-*tert*-butyl-4-hydroxytoluene (BHT) enhances complex formation between full-length CYP2B4 and full-length cyt *b*_5_ in a membrane mimetic environment^24^. Crystal structures of P450s have demonstrated that substrate binding causes conformational changes on the proximal surface of P450 where the redox partners bind^25^,^26^,^27^,^28^,^29^,^30^,^31^,^32^,^33^,^34^,^35^. A more detailed look into the cyt *b*_5_-P450 complex formation process under the influence of substrates, in a native-like environment, will lead to a better understanding of the role that substrates play in modulating the interactions between these two redox partners.

In this study, NMR techniques are used to investigate the interactions, at the atomic level, between full-length substrate-free/BHT-bound CYP2B4 and full-length rabbit cyt *b*_5_ in a membrane mimetic environment under different ionic strength conditions. By titrating NaCl into the complexes, we explored the important role of electrostatic interactions in the cyt *b*_5_-CYP2B4 complex formation. We found that varying the ionic strength affected complex formation between cyt *b*_5_-substrate-free CYP2B4 and cyt *b*_5_-BHT-bound CYP2B4 in different ways, revealing different encounter/stereospecific complex population ratios in the two cases. The findings unveil the role that the substrate BHT plays in the complexation process. Furthermore, a detailed analysis of the differential ionic strength dependence of resonance intensities of cyt *b*_5_ residues allowed for identification of residues most likely involved in specific interactions with CYP2B4, leading to a better understanding of the complex interface.

## Results

### NaCl does not affect the structures of cyt b_5_ and CYP2B4

Through the use of Circular Dichroism (CD) experiments, in which NaCl was titrated into CYP2B4 incorporated in isotropic bicelles composed of DMPC (1,2-dimyristoyl-*sn*-glycero-3-phosphocholine) and DHPC (1,2-dihexanoyl-*sn*-glycero-3-phosphocholine) (*q* = 0.25), we confirm that the overall secondary structure of CYP2B4 does not change with the addition of NaCl (Fig. S1). This is in agreement with a previous study which showed no secondary structure changes in CYP2B4 at different sodium phosphate and NaCl concentrations^40^.

To assess whether the structure of cyt *b*_5_, incorporated in DMPC/DHPC isotropic bicelles (*q* = 0.25), is affected upon the addition of NaCl, a series of 2D ^1^H/^15^N-SOFAST-HMQC (band-Selective Optimized-Flip-Angle Short-Transient Heteronuclear Multiple Quantum Correlation) spectra of cyt *b*_5_ were recorded during NaCl titration. The spectra reveal no significant structural changes of cyt *b*_5_ since only slight to negligible chemical shift perturbations are observed (average perturbation < 0.009 ppm) (Fig. S2). In addition, no significant perturbations in signal intensities are observed (average intensity change of 2.3% relative to free cyt *b*_5_ intensities) except for the N-terminal loop region (K7 and D8), residues S93 to D104 in the linker region and the residue E48. All of these residues show intensity changes of more than one standard deviation away from the average change and are therefore not included in the data analysis of the salt titration on the cyt *b*_5_-CYP2B4 complexes. These residues are highly solvent-exposed and the perturbations they experience are likely due to changes in buffer conditions. Addition of BHT to cyt *b*_5_ also causes no spectral changes, neither in intensities nor chemical shifts (Fig. S3).

### Interaction between cyt b_5_ and substrate-free CYP2B4 during NaCl titration

In order to study the influence of ionic strength on the interaction between cyt *b*_5_ and substrate-free CYP2B4, cyt *b*_5_-CYP2B4 complexes were first formed and incorporated in DMPC/DHPC bicelles (*q* = 0.25), and then titrated with NaCl. A series of 2D ^1^H/^15^N-TROSY-HSQC (Transverse Relaxation Optimized SpectroscopY Heteronuclear Single Quantum Correlation) spectra were recorded at the following NaCl concentrations: 0, 100, 250 and 400 mM.

**Figure 1:**
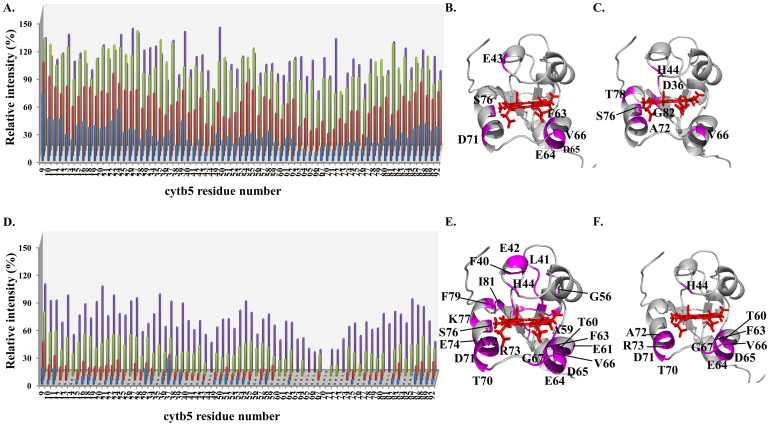
Relative intensity recovery of cyt *b*_5_ resonances in complex with substrate-free (A–C)/BHT-bound (D-F) CYP2B4 during NaCl titration. Relative intensities of backbone amides of cyt *b*_5_ in a 1:1 molar ratio with CYP2B4, represented as a percentage of the corresponding resonance intensity in free cyt *b*_5_, are plotted for each cyt *b*_5_ residue for (A) the complex between cyt *b*_5_ and substrate-free CYP2B4 and (D) the complex between cyt *b*_5_ and BHT-bound CYP2B4 at the following NaCl concentrations: 0 mM (blue), 100 mM (red), 250 mM (green) and 400 mM (purple). Residues with relative intensities of more than one standard deviation below the average are mapped onto the cyt *b*_5_ structure (magenta) before and after NaCl titration: cyt *b*_5_-substrate-free CYP2B4 at 0 mM (B) and 400 mM (C) NaCl; and cyt *b*_5_-BHT-bound CYP2B4 at 400 mM NaCl (F). All the residues that broadened beyond detection for the cyt *b*_5_-BHT-bound CYP2B4 complex at 0 mM NaCl are shown in (E), but mapping of these residues does not provide information on the specific binding interface of cyt *b*_5_-BHT-bound CYP2B4 (see Results). However, it demonstrates the remarkable line-broadening of cyt *b*_5_ upon complexation with BHT-bound CYP2B4. The cyt *b*_5_ structure in these figures is the NMR-derived structure previously reported (PDB code: 2M33)^24^.

When CYP2B4 and ^15^N-cyt *b*_5_ form a 1:1 complex, general broadening of resonances (Fig. 1A, blue) and modest chemical shift perturbation (CSP) (average < 0.01 ppm) are observed for cyt *b*_5_ backbone amides. These two findings suggest the interaction takes place on a fast-to-intermediate time scale (ns - µs)^23^,^24^. The relative intensities of each cyt *b*_5_ backbone amide resonance, represented as a percentage of the corresponding resonance intensities in the ^1^H/^15^N-TROSY-HSQC spectrum of free cyt *b*_5_ (see Materials and Methods), are plotted against cyt *b*_5_ residue number for each NaCl titration point in Figure 1A. When cyt *b*_5_ interacts with one molar equivalent of substrate-free CYP2B4 at 0 mM NaCl, the average relative intensity of cyt *b*_5_ resonances drops down to 34.1% of that observed in the free cyt *b*_5_ spectrum. Residues significantly affected (with a relative intensity more than one standard deviation below the average) upon complex formation with CYP2B4 include E43, F63, E64, D65, V66, D71 and S76. These residues are located on the proximal side of cyt *b*_5_ where the heme is solvent-exposed (Fig. 1B), and are consistent with our previous findings^24^.

**Figure 2:**
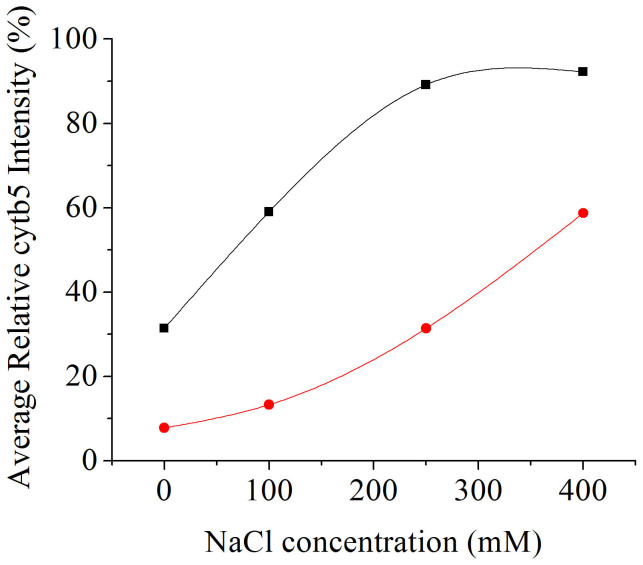
Ionic strength dependence of the average relative intensity of cyt *b*_5_ resonances in the cyt *b*_5_-substrate-free CYP2B4 complex (black) and cyt *b*_5_-BHT-bound CYP2B4 complex (red). The black curve reaches a plateau at 250 mM NaCl (the third data point) while the red curve still increases even at 400 mM NaCl (the last data point). The first three data points (before reaching the plateau) of the cyt *b*_5_-substrate-free CYP2B4 complex (black) increase linearly with a slope of 0.23 and R^2^ value of 0.99. The cyt *b*_5_-BHT-bound CYP2B4 complex data points (red) increase linearly with a slope of 0.12 and R^2^ value of 0.96. Excel was used to fit the linear trend lines.

**Figure 3:**
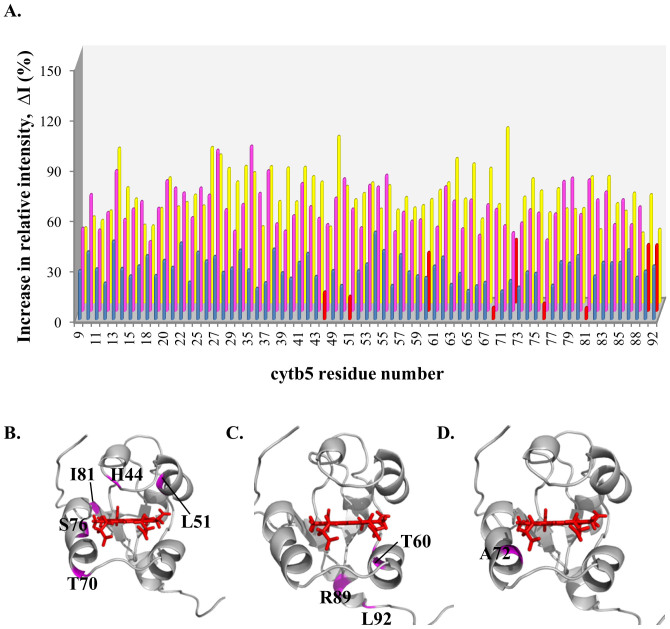
Mapping of cyt *b*_5_ residues minimally affected by the increasing ionic strength when in complex with substrate-free CYP2B4. (A) Relative intensity increase (ΔI) of cyt *b*_5_ resonances at 100 mM (blue), 250 mM (magenta) and 400 mM (yellow) NaCl, each calculated relative to 0 mM NaCl (*e.g.* ΔI for 250 mM NaCl = I_250mM NaCl_ – I_0mM NaCl_). Residues with ΔI more than one standard deviation below the average are considered insignificantly affected by the ionic strength increment. These residues are highlighted in red in the histogram in (A), and are mapped in magenta onto the cyt *b*_5_ structure for 100 mM NaCl (B), 250 mM NaCl (C) and 400 mM NaCl (D). No common residue is found in B, C and D.

NaCl was subsequently titrated into the cyt *b*_5_-CYP2B4 complex and a general increase in relative resonance intensities was observed with increasing NaCl concentration (Fig. 1A). The average relative intensities of cyt *b*_5_ resonances at 0, 100, 250 and 400 mM NaCl are 31.4, 59.1, 89.2 and 92.2% respectively and start to reach a plateau at 250 mM NaCl (Fig. 2, black). The first three titration points (before reaching the plateau) could be fit using a linear trend line with a slope of 0.23 (R^2^ = 0.99), which approximately represents the rate of intensity increase with respect to NaCl concentration. To assess how individual residues are influenced by ionic strength, the relative intensities of the cyt *b*_5_ resonances at each NaCl titration step are also compared to those at 0 mM NaCl to determine the change in intensity, ΔI (see Materials and Methods), and plotted for each cyt *b*_5_ residue (Fig. 3A). The average intensity difference values (ΔI_avg_) for each NaCl concentration are listed in Table 1. By identifying residues with a ΔI more than one standard deviation below ΔI_avg_, certain cyt *b*_5_ residues can be identified as being insignificantly affected upon salt addition. These residues include H44, L51, T70, S76 and I81 at 100 mM NaCl (Fig. 3B); T60, R89 and L92 at 250 mM NaCl (Fig. 3C); and A72 at 400 mM NaCl (Fig. 3D). It can be seen that no residue is found to maintain weak salt dependence throughout the entire NaCl titration. At 400 mM NaCl, however, certain cyt *b*_5_ residues still remain relatively low in intensity (with a relative intensity more than one standard deviation below the average relative intensity) and include D36 and G82 on the back of cyt *b*_5_, as well as H44, V66, A72, S76 and T78 on the upper and lower cleft of cyt *b*_5_, around the heme edge (Fig. 1C).

### Interaction between cyt b_5_ and substrate-bound CYP2B4 during NaCl titration

We previously showed that the interaction between cyt *b*_5_ and CYP2B4 is altered by the presence of a CYP2B4 substrate^24^. To understand the changes in the protein-protein interaction caused by a substrate, the effect of ionic strength on the 1:1 full-length complex between cyt *b*_5_ and CYP2B4, incorporated in DMPC/DHPC isotropic bicelles (*q* = 0.25), was studied in the presence of the CYP2B4 type I substrate 3,5-di-*tert*-butyl-4-hydroxytoluene (BHT). The complex between cyt *b*_5_ and BHT-bound CYP2B4 was monitored during the titration of 0, 100, 250 and 400 mM NaCl with a series of two-dimensional ^1^H/^15^N-TROSY-HSQC spectra.

As shown in Figure 1D (blue), formation of the complex between cyt *b*_5_ and BHT-bound CYP2B4 leads to extensive line-broadening of ^15^N-labeled cyt *b*_5_ amide resonances, among which ~50% of the residues disappear from the spectrum (Fig. 1D, blue), which suggests intermediate-to-slow chemical exchange between free- and bound-cyt *b*_5_^24^. All cyt *b*_5_ residues that disappear from the spectrum are mapped onto the cyt *b*_5_ structure in Figure 1E. However, due to the disappearance of the majority of cyt *b*_5_ resonances from the spectrum, the interaction interface on cyt *b*_5_ cannot be accurately defined. The majority of the residues that broaden beyond detection upon the addition of BHT-bound CYP2B4 are located on the proximal side of cyt *b*_5_, surrounding the solvent-exposed heme edge. The more significant line-broadening of cyt *b*_5_ resonances observed when cyt *b*_5_ interacts with BHT-bound CYP2B4, as compared to its interaction with substrate-free CYP2B4, suggests tighter binding between the two proteins as a result of the presence of the substrate BHT^24^.

**Figure 4:**
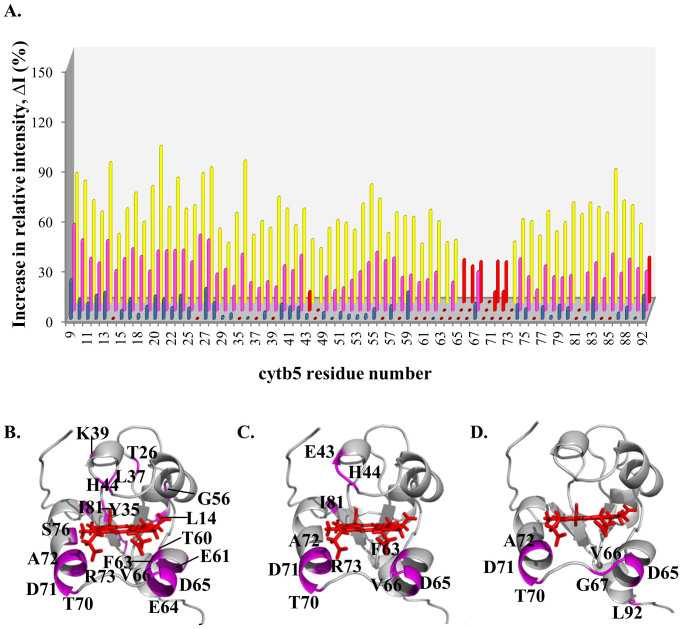
Mapping of cyt *b*_5_ residues minimally affected by the increasing ionic strength when in complex with BHT-bound CYP2B4. (A) Increase in relative intensities (ΔI) of cyt *b*_5_ resonances at 100 mM (blue), 250 mM (magenta) and 400 mM (yellow) NaCl, each calculated relative to 0 mM NaCl. Residues with ΔI more than one standard deviation below the average are highlighted in red in the histogram in (A), and are mapped in magenta onto the cyt *b*_5_ structure for 100 mM NaCl (B), 250 mM NaCl (C) and 400 mM NaCl (D). The following residues are found to be highlighted in all the three figures (B, C and D): D65, V66, T70, D71 and A72. These residues are most likely involved in specific interactions during complex formation.

Subsequent titration of NaCl causes an increase in relative intensities of cyt *b*_5_ resonances (Fig. 1D). The average relative intensities at 0, 100, 250 and 400 mM NaCl are 7.78, 13.3, 31.3 and 58.7% respectively. Unlike in the substrate-free complex, the average relative intensities of cyt *b*_5_ resonances in the CYP2B4 BHT-bound complex show no sign of reaching a plateau at the highest ionic strength of 400 mM NaCl (Fig. 2, red). The four data points fit a linear trend line with a slope of 0.12 (R^2^ = 0.96), which approximately represents the rate of relative intensity increase as a function of NaCl concentration. Residue-wise relative intensity increase (ΔI) and the average intensity increase (ΔI_avg_) observed at each titration step, relative to 0 mM NaCl, are plotted in Figure 4A and listed in Table 1 respectively. At 100 mM NaCl, twenty-two cyt *b*_5_ residues can be identified as having insignificant relative intensity increase (ΔI more than one standard deviation below ΔI_avg_), the majority of which are located on the proximal side of cyt *b*_5_ around the heme edge (Fig. 4B). At 250 mM NaCl, residues exhibiting insignificant ΔI include E43, H44, F63, D65, V66, T70, D71, A72, R73 and I81 (Fig. 4C). At 400 mM NaCl, residues D65, V66, G67, T70, D71, A72 and L92 are observed to show insignificant ΔI (Fig. 4D). The following residues therefore demonstrate weak dependence on NaCl concentration throughout the entire titration: D65, V66, T70, D71 and A72. These five residues are located on the proximal side of cyt *b*_5_, surrounding the heme edge, and were previously hypothesized to be at the binding interface with CYP2B4^24^. At 400 mM NaCl, certain cyt *b*_5_ residues remain significantly line-broadened (relative intensity more than one standard deviation below the average relative intensity): H44 on the upper cleft of cyt *b*_5_, as well as T60, F63, E64, D65, V66, G67, T70, D71, A72 and R73 on the lower cleft of cyt *b*_5_, around the heme edge (Fig. 1F).

## Discussion

### Cyt b_5_-CYP2B4 complex population decreases with increasing ionic strength

**Figure 5:**
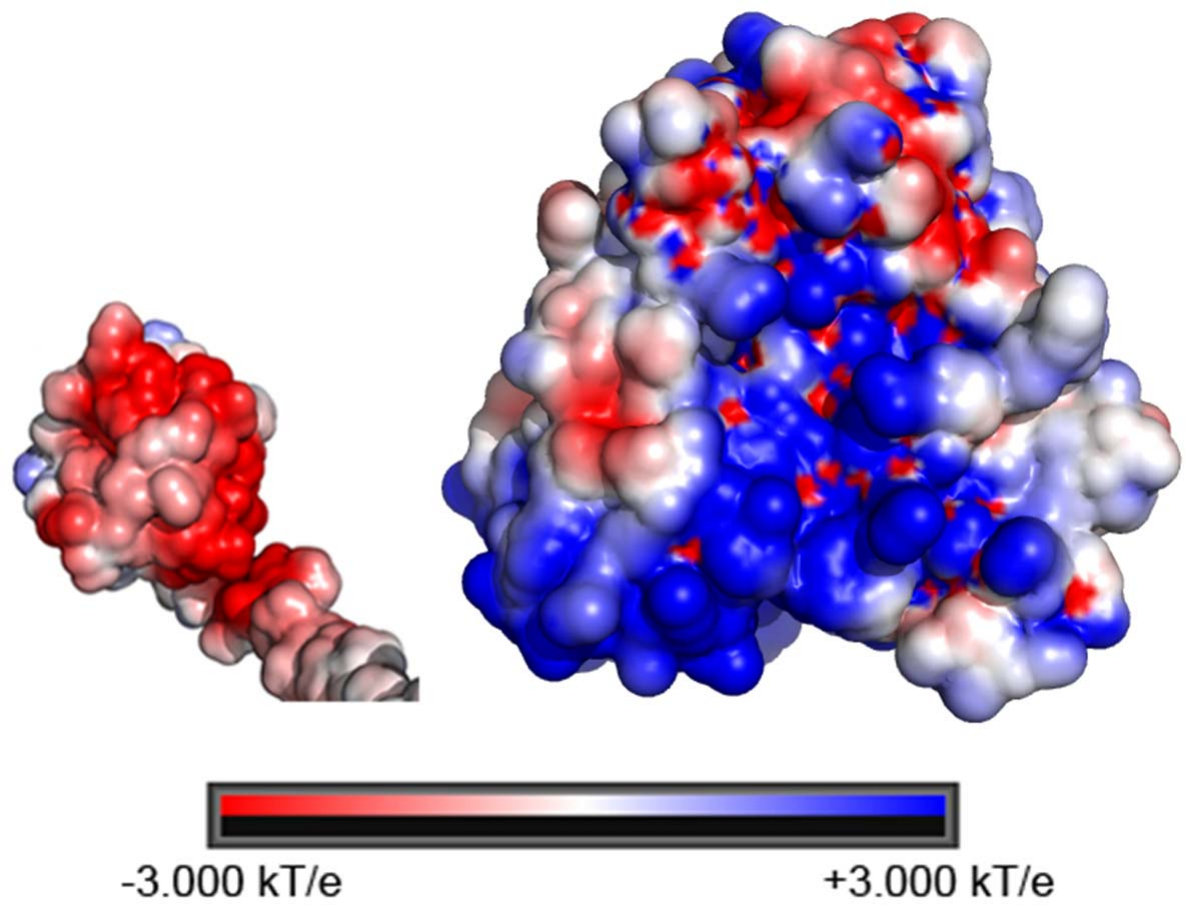
Electrostatic potential surfaces of the proximal sides of cyt *b*_5_ (left) and CYP2B4 (right), showing the negatively charged convex surface of cyt *b*_5_ (PDB code: 2M33)^24^ and the positively charged concave surface of CYP2B4 (PDB code: 1SUO^34^). The electrostatic potential surfaces were calculated using the PDB2PQR^50^,^51^ server and PyMOL^52^ with the APBS plugin 2.1^53^.

Our results reveal that complex formation between cyt *b*_5_ and CYP2B4, both substrate-free and BHT-bound, is ionic strength dependent. The broadening of ^15^N-labeled cyt *b*_5_ amide resonances observed in the ^1^H/^15^N-TROSY-HSQC spectrum upon complexation with CYP2B4 can be reversed by the introduction of NaCl into the system (Fig. 1A, Fig. 1D and Fig. 2), implying liberation of free cyt *b*_5_ and dissociation of the complex. These observations are indicative of the importance of the attractive electrostatic interactions in complex formation. This is not surprising given the net charges of the two proteins at pH 7.4: +6.9 for CYP2B4 and -8.4 for cyt *b*_5_. The opposite electrostatic surface potentials of the two proteins (Fig. 5) could promote favorable electrostatic docking between the two proteins. Long-range electrostatic interactions are known to facilitate protein-protein complexation in two ways^14^,^15^,^41^: first, Coulombic attraction between oppositely charged surfaces, like the acidic convex surface of cyt *b*_5_ and the concave basic proximal side of CYP2B4 (Fig. 5), keeps the two proteins in close proximity and lengthens the lifetime of the macro-collision, enabling more extensive surface sampling through translational and rotational movements; second, pre-orientation of the two proteins is steered by the interactions between complimentary charged patches of residues on each protein, which results in a significant reduction of the searching area and increases the probability of reaching a productive orientation in which electron transfer can occur. Therefore, the salt screening effect caused by high ionic strength should, to some extent, hinder the part of complex formation in which electrostatic interactions play an important role. Similar results have been observed for a number of other protein complexes, where the complex association constant decreases with increasing ionic strength, especially for complexes between redox partners, *e.g.* cytochrome f and cytochrome c_6_^42^, Ana-PSI and Ana-Pc^43^, and barnase and barstar^13^,^44^.

### Substrates promote specific interactions between CYP2B4 and cyt b_5_

As mentioned, it has been proposed that macromolecular association can be divided into two steps^11^,^13^,^15^,^16^,^45^: first, the formation of an ensemble of encounter complexes through three dimensional random collisions of the two molecules, in which long-range non-specific electrostatic interactions have been suggested to play an important role by increasing the collision rate constants and reducing the searching dimensionality on the surfaces of the binding partners; second, subsequent rotations and translations along each other's surfaces rearrange the two molecules to satisfy the stringent orientational requirements for specific interactions, which leads to the formation of the final well-defined stereospecific complex(es). Because the interfaces of encounter complexes are much less compact than that of stereospecific complex(es), ions have easier access to the encounter complexes' interfaces and the salt screening effect of interfacial charges for encounter complexes is more significant than for the stereospecific complex(es), upon an increase in ionic strength^16^. For these reasons, encounter complexes have been found to be more sensitive to ionic strength than the stereospecific complex(es)^16^.

In the present study, the dependence of complex formation on ionic strength is investigated for both cyt *b*_5_-substrate-free CYP2B4 and cyt *b*_5_-BHT-bound CYP2B4 complexes. The average relative cyt *b*_5_ resonance intensity increase (ΔI_avg_) at each NaCl titration step are listed in Table 1 for both cases. It is apparent that the average relative intensity increase is greater for the cyt *b*_5_-substrate-free CYP2B4 complex than for the cyt *b*_5_-BHT-bound CYP2B4 complex when adding 100 mM (> 5x greater) and 250 mM (> 2x greater) NaCl. At 400 mM NaCl, however, the intensity increase is similar in the two cases, with cyt *b*_5_-substrate-free CYP2B4 being slightly bigger (ΔI_avg_ of 60% versus 50%), which is a result of the fact that saturation in intensity is reached at 250 mM NaCl in the case of cyt *b*_5_-substrate-free CYP2B4 (average relative intensity of 92.2%) while the relative intensity is still increasing for cyt *b*_5_-BHT-bound CYP2B4 (average relative intensity of 58.7%) (Fig. 2). By looking at Figure 2 and comparing the slopes of the linear portions for each complex type, a higher dependence on salt concentration is observed when CYP2B4 is substrate-free (slope of 0.23 for cyt *b*_5_-substrate-free CYP2B4 and 0.12 for cyt *b*_5_-BHT-bound CYP2B4). These observations indicate that the complex between cyt *b*_5_ and CYP2B4 is more sensitive to ionic strength in the absence of a substrate than when CYP2B4 is bound to the substrate BHT. Considering that stereospecific complexes have been shown to be affected by ionic strength to a smaller degree than encounter complexes^16^, our results thereby suggest that there is a larger fraction of stereospecific complexes for cyt *b*_5_-BHT-bound CYP2B4 than when CYP2B4 is substrate-free. In other words, the substrate BHT likely promotes specific interactions between cyt *b*_5_ and CYP2B4, causing a higher population ratio of stereospecific complex(es) to encounter complexes. This might be due to a conformational change of CYP2B4 in order to accommodate BHT. Crystal structures of CYP2B4 have demonstrated ligand-induced structural rearrangements of the protein when bound to diverse substrates^25^,^26^,^27^,^28^,^29^,^30^,^31^,^32^,^33^,^34^,^35^, and have highlighted five plastic regions (PR1-5) subject to conformational variability; PR2 includes the C helix and the C-D loop region on the proximal side of CYP2B4^46^. Interestingly, mutagenesis studies have shown that six out of seven interface residues on CYP2B4, when complexed with cyt *b*_5_, are on the C helix and the C-D loop^37^. Our previously reported complex structure of cyt *b*_5_-CYP2B4 also showed that the binding interface on CYP2B4 was predominantly located on the C helix and the C-D loop^24^. Therefore, a possible explanation for the capability of BHT to promote specific interactions during cyt *b*_5_-CYP2B4 complex formation could be that a conformational rearrangement of the CYP2B4 C helix and C-D loop region is induced upon accommodation of BHT; this might place the key binding sites on CYP2B4 into specific orientations such that favorable contacts with the binding interface on cyt *b*_5_ can occur, thus leading to a shift in equilibrium from the encounter complexes to the stereospecific complex(es).

Since short-range specific interactions are much stronger than long-range non-specific electrostatic interactions, this shift towards the stereospecific complexes also explains our observation that interactions between cyt *b*_5_ and BHT-bound CYP2B4 lead to more extensive line-broadening in the NMR spectrum, implying higher affinity between the two proteins, than that observed for cyt *b*_5_-substrate-free CYP2B4 (Figs. 1A and 1D, blue)^24^. Additionally, at 400 mM NaCl (the highest ionic strength tested in our experiments), the average relative intensity for cyt *b*_5_ in complex with substrate-free CYP2B4 returns to almost 100% (Fig. 2, black), and the residues that remain relatively low in intensity, compared to other residues, are spread over an extensive area on cyt *b*_5_ (Fig. 1C), suggesting very weak (nearly negligible) and non-specific interactions between the two proteins at this ionic strength. These two observations also suggest that cyt *b*_5_-substrate-free CYP2B4 predominantly comprises encounter complexes. The salt screening effect caused by high ionic strength should thus lead to a markedly reduced lifetime of these encounter complexes^47^,^48^, thereby leaving insufficient time for the necessary re-orientation of the two partners that would allow for the formation the well-defined stereospecific complex(es)^15^. For cyt *b*_5_-substrate-free CYP2B4, the probability of reaching the stereospecific complex(es) is therefore greatly reduced with increasing ionic strength, and this explains the observation that the cyt *b*_5_-substrate-free CYP2B4 complexes are nearly all dissociated at 400 mM NaCl. In contrast, at the same ionic strength (400 mM NaCl), cyt *b*_5_-BHT-bound CYP2B4 still exhibits ~50% reduction in intensity (on average) relative to free cyt *b*_5_ intensities (Fig. 2, red) and a very localized binding interface on cyt *b*_5_ (Fig. 1F), indicating high affinity and specificity even at 400 mM NaCl. Since the stereospecific complexes are affected by ionic strength to a lesser extent than encounter complexes, this observation could be attributed to a higher population ratio of stereospecific/encounter complexes due to the equilibrium shift induced by BHT, as mentioned above. By looking at the binding interface observed at the titration point of 400 mM NaCl (Fig. 1F), we should therefore be able to better define the stereospecific interaction interface of cyt *b*_5_.

### Identification of cyt b_5_ residues involved in specific interactions with CYP2B4

Cyt *b*_5_ residues hardly perturbed by ionic strength changes are likely to be involved in short-range specific interactions with CYP2B4 due to the relatively more closely packed binding interface of the stereospecific complexes^16^. Following the relative intensity increase, ΔI, for each cyt *b*_5_ residue, at each step of NaCl titration into cyt *b*_5_-BHT-bound CYP2B4, we are able to narrow down the number of residues that are most likely involved in specific interactions with CYP2B4 to D65, V66, T70, D71 and A72. These residues experience small changes in intensity (ΔI more than one standard deviation below ΔI_avg_) with increasing ionic strength throughout the entire titration (Fig. 4). These five residues were previously found at the interface of the cyt *b*_5_-CYP2B4 complex generated by docking studies, with D65 and D71 forming hydrogen bonds and salt bridges with K433/R122 and D134/R133 on CYP2B4 respectively, and V66, T70 and A72 forming van der Waals interactions with K433/R125, A130/D134/G136 and R126 on CYP2B4 respectively^24^. D65 and V66 have also been shown by a mutagenesis study to be essential for the formation of productive complexes between cyt *b*_5_ and CYP2B4^24^. Although our identification of D71 as a likely interfacial residue is discrepant with published mutagenesis results demonstrating D71 to be unimportant for cyt *b*_5_ binding to CYP2B4^24^, it is not uncommon for interfacial residues to contribute insignificant free energy of binding^49^. In contrast to cyt *b*_5_-BHT-bound CYP2B4, no residue maintained weak salt dependence throughout the NaCl titration of cyt *b*_5_-substrate-free CYP2B4 (Fig. 3), which could be attributable to the more pronounced encounter complex character of this complex, due to the larger encounter complex population.

In conclusion, our study demonstrates the importance of electrostatic interactions in complex formation between cyt *b*_5_ and CYP2B4, both substrate-free and BHT-bound, as indicated by the ionic strength dependence of cyt *b*_5_ resonance intensities. A comparison of the ionic strength dependence between cyt *b*_5_-substrate-free CYP2B4 and cyt *b*_5_-BHT-bound CYP2B4 reveals a higher ratio of stereospecific to encounter complex populations when CYP2B4 is BHT-bound. This finding suggests the ability of BHT to promote specific interactions between the two proteins, likely through conformational modulation of CYP2B4, which might lead to more efficient electron transfer and substrate turn-over. Additionally, five cyt *b*_5_ residues, namely D65, V66, T70, D71 and A72 are identified to be most likely involved in specific interactions with CYP2B4, providing structural insights into the interactions between cyt *b*_5_ and BHT-bound CYP2B4.

## Methods

### Materials

Phosphate buffer components (potassium phosphate monobasic and dibasic), glycerol and 3,5-di-*tert*-butyl-4-hydroxytoluene (BHT) were purchased from Sigma-Aldrich (St. Louis, MO). Sodium chloride was purchased from Fisher Scientific. 1,2-dihexanoyl-*sn*-glycero-3-phosphocholine (DHPC) and 1,2-dimyristoyl-*sn*-glycero-3-phosphocholine (DMPC) were purchased from Avanti Polar Lipids, Inc. (Alabaster, AL).

### NMR experiments

Full-length ^15^N-labeled cyt *b*_5_ and unlabeled full-length CYP2B4 were expressed and purified as reported previously^36^,^37^. NMR experiments were performed at 298 K on a Bruker 900 MHz spectrometer equipped with a 5 mm triple-resonance TXI cryo-probe. All NMR samples were prepared in 100 mM potassium phosphate buffer, pH 7.4, with 5% (w/v) of glycerol. DMPC/DHPC isotropic bicelles (*q* = [DMPC]/[DHPC] = 0.25) were prepared by co-solubilizing DMPC and DHPC in chloroform. The solvent was then evaporated with N_2_ gas to form a thin film at the bottom of a test tube; any residual chloroform was completely removed by lyophilizing in a vacuum oven overnight. The lipid film was rehydrated prior to preparing the NMR sample^38^. The final concentration of bicelles in all NMR samples was 10% (w/v).

The NaCl titration experiments on cyt *b*_5_ alone were done on 0.2 mM ^15^N-cyt *b*_5_ incorporated in DMPC/DHPC isotropic bicelles (*q* = 0.25). A series of ^1^H/^15^N-SOFAST-HMQC spectra of ^15^N-cyt *b*_5_ were collected under the following four conditions: 0, 100, 250 and 400 mM NaCl.

For studies of the cyt *b*_5_-CYP2B4 complex, complexes of cyt *b*_5_ and CYP2B4 were prepared with 0.2 mM concentration of each protein and incorporated in 10% (w/v) DMPC/DHPC isotropic bicelles (*q* = 0.25). The complex samples were titrated with NaCl and monitored by recording ^1^H/^15^N-TROSY-HSQC spectra. The complex samples containing BHT had a molar ratio of 2:1 of BHT to CYP2B4. NaCl titrations were performed on both the protein complex samples with and without BHT. The titrations were carried out with 0, 100, 250 and 400 mM NaCl.

A ^1^H/^15^N-TROSY-HSQC reference spectrum of 0.1 mM ^15^N-labeled cyt *b*_5_ incorporated in 10% (w/v) DMPC/DHPC isotropic bicelles (*q* = 0.25) was also recorded (referred to as “free cyt *b*_5_” in the text). The free cyt *b*_5_ backbone amide resonance peak heights from this spectrum were used to calculate the relative intensities of each cyt *b*_5_ residue when in complex with CYP2B4, under the various conditions tested. For this calculation, the free cyt *b*_5_ resonance peak heights were taken as 100% and the peak heights in complex with CYP2B4 were taken as a percentage of the free cyt *b*_5_ peak heights, referred to as relative intensities, in all analyses. For the spectra in which certain cyt *b*_5_ resonances were not visible in the spectrum (where the resonances broadened beyond detection), a relative intensity of 0% was assigned to these residues.

All spectra were processed in TopSpin 2.0. Peak assignments were accomplished using Sparky, and chemical shifts as well as peak heights were also obtained from Sparky^39^. Assignment of resonances from the soluble domain of ferric cyt *b*_5_ in the ^1^H/^15^N-TROSY-HSQC spectra was previously reported^24^.

To calculate ΔI, the difference between the relative intensity of cyt *b*_5_ at a given ionic strength and 0 mM NaCl was taken (*e.g.* ΔI for 100 mM NaCl = I_100mM NaCl_ – I_0mM NaCl_). The value ΔI_avg_ is the average of ΔI values over all cyt *b*_5_ residues at that ionic strength.

### Circular dichroism (CD) experiments of CYP2B4

CD experiments were performed on a Jasco J-715 spectropolarimeter fitted with a 150-W xenon lamp at 25°C using a 1 mm cuvette. Bandwidth was set at 1.0 nm and the time constant was 1.0 s. Spectra were recorded in the far UV region from 190 to 270 nm, with 8 scans accumulated and averaged for each spectrum. Background (with everything present except CYP2B4) was subtracted for all experiments. NaCl was titrated into a solution containing 1 µM CYP2B4 and 2% (w/v) DMPC/DHPC bicelle (*q* = 0.25) in 100 mM potassium phosphate buffer, pH 7.4, containing 5% (w/v) glycerol. The following concentrations of NaCl were tested: 1 µM, 250 µM, 500 µM, 675 µM, 1 mM, 1.25 mM and 2 mM. These concentrations cover the molar ratios of CYP2B4 to NaCl that are equivalent to those in the NMR samples.

## Author Contributions

M.Z., S.V.L, R.H., S.A., S.I., L.W. and A.R. performed the experiments. M.Z., S.V.L, R.H. and A.R. analyzed the results and wrote the paper. A.R. designed and directed the research. All authors reviewed the manuscript.

## Supplementary Material

Supplementary Information Supporting information

## Figures and Tables

**Table 1:** Change of the average relative intensities (ΔI_avg_) of cyt *b*_5_ resonances at each NaCl concentration relative to those at 0 mM NaCl

[NaCl] (mM)	ΔI_avg_ (%) in the absence of BHT	ΔI_avg_ (%) in the presence of BHT
100	27.6	5.5
250	57.8	23.6
400	60.8	50.9
