# Supplementary material for: Insights into the Role of Substrates on the Interaction between Cytochrome b5 and Cytochrome P450 2B4 by NMR
Source: Sci Rep. 2015 Feb 17;5:8392. doi: 10.1038/srep08392 (PMC4330534; doi:10.1038/srep08392)
Supplement: Supplementary Information — Supporting information [file srep08392-s1.pdf]

Supporting information

Insights into the Role of Substrates on the Interaction between Cytochrome b_5 and Cytochrome P450 2B4 by NMR

Meng Zhang[†1], Stéphanie V. Le Clair[†1], Rui Huang[†], Shivani Ahuja[†], Sang-Choul Im[‡], Lucy Waskell[‡] and Ayyalusamy Ramamoorthy[†]

[†]Department of Chemistry and Biophysics, University of Michigan, Ann Arbor, Michigan 48109-1055, USA

[‡]Department of Anesthesiology, University of Michigan, and VA Medical Center, Ann Arbor, Michigan 48105, USA

To whom the correspondence should be addressed: Ayyalusamy Ramamoorthy, Department of Chemistry and Biophysics, University of Michigan, Ann Arbor, Michigan 48109-1055, USA, Tel.: (734) 647-6572; Fax: (734) 764-3323; E-mail: ramamoor@umich.edu

FIGURE LEGENDS

FIGURE S1. The secondary structure of CYP2B4 is not affected upon the addition of NaCl. Circular Dichroism spectra of CYP2B4, incorporated in 2% (w/v) DMPC/DHPC isotropic bicelles (*q* = 0.25), collected before and during titration of NaCl.

FIGURE S1. **The overall structure of cyt b_5 is not affected by the addition of NaCl**. $^{1}H/^{15}N$-SOFAST-HMQC spectra of ^{15}N-cyt b_5, incorporated in DMPC/DHPC isotropic bicelles (*q* = 0.25), collected at the indicated salt conditions.

FIGURE S2. Addition of BHT affects neither intensities nor chemical shifts of cyt b_5 backbone NH resonances. The $^{1}H/^{15}N$-SOFAST-HMQC spectrum of ^{15}N-cyt b_5 in isotropic bicelles (maroon) and the spectrum obtained after addition of BHT in a 2:1 molar ratio of BHT to cyt b_5 (coral) superimposed above it.

FIGURE S1.

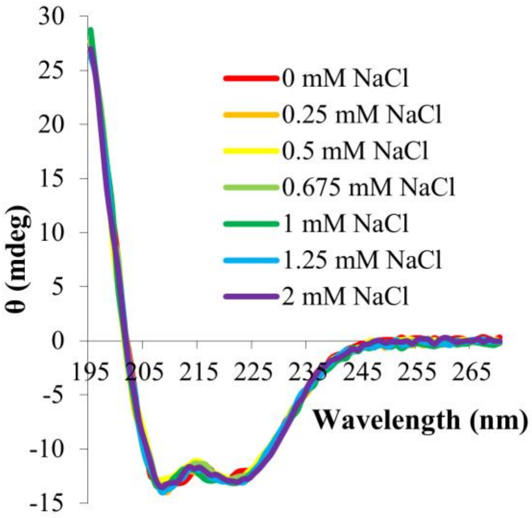

0 mM NaCl
0.25 mM NaCl
0.5 mM NaCl
0.675 mM NaCl
1 mM NaCl
1.25 mM NaCl
2 mM NaCl
θ (mdeg)
Wavelength (nm)
30
25
20
15
10
5
0
-5
-10
-15
195
205
215
225
235
245
255
265

FIGURE S2

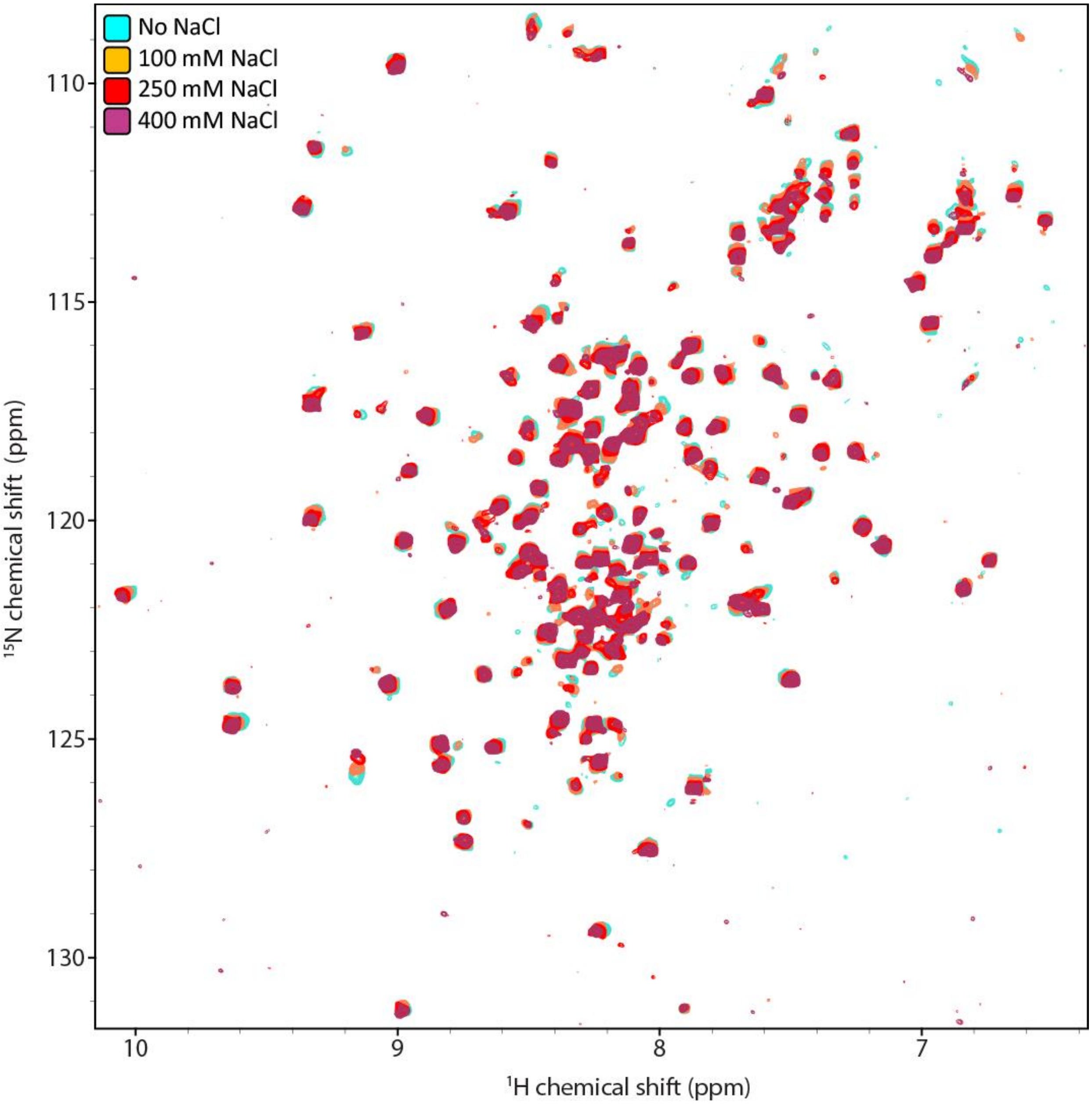
No NaCl
100 mM NaCl
250 mM NaCl
400 mM NaCl
110
115
120
125
130
^{15}N chemical shift (ppm)
10
9
8
7
^{1}H chemical shift (ppm)

FIGURE S3

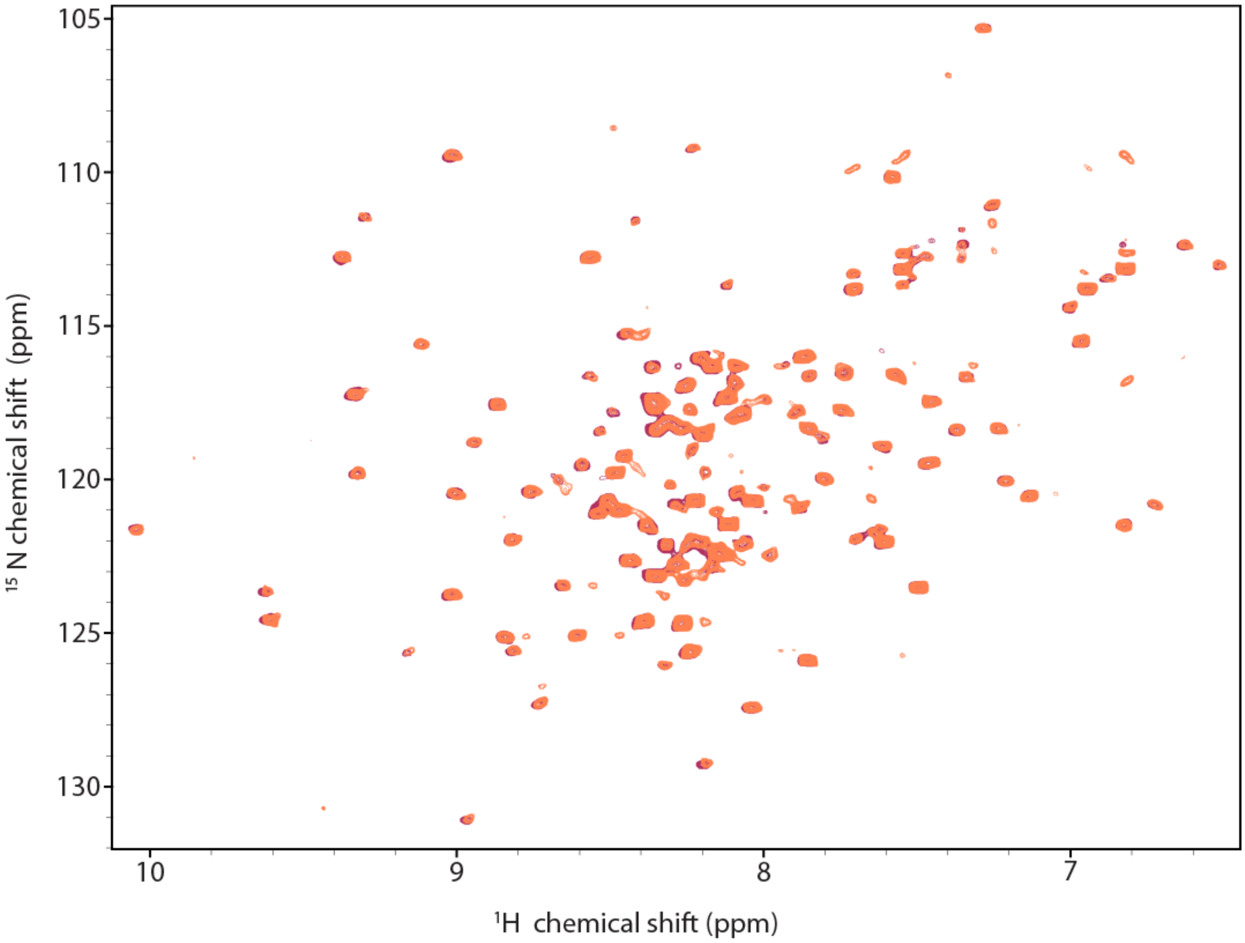

105
110
115
120
125
130
15 N chemical shift (ppm)
10
9
8
7
1H chemical shift (ppm)
